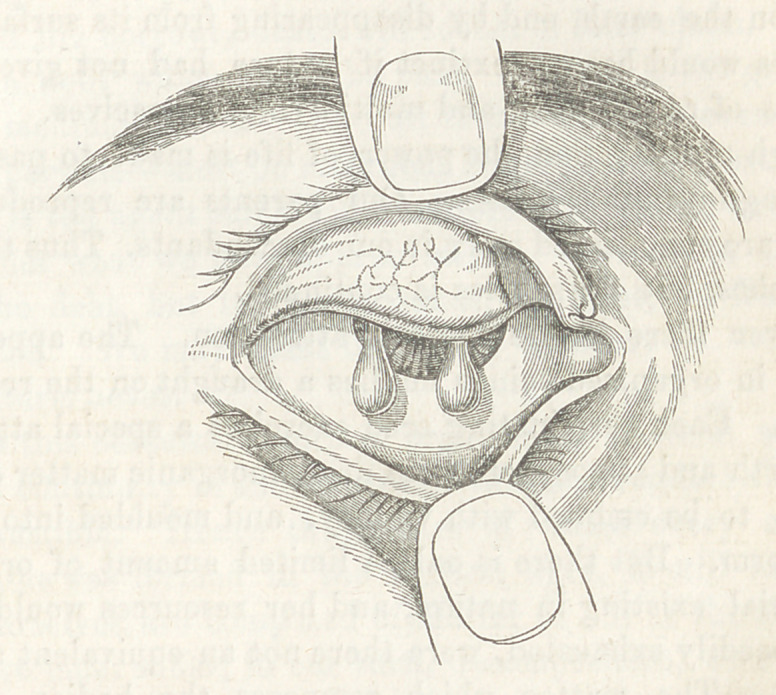# A Case of Polypi of the Conjunctiva

**Published:** 1855-04

**Authors:** John Neill

**Affiliations:** Surgeon to the Pennsylvania Hospital


*A Case of Polypi of the Conjunctiva.* By JOHN NEILL, M. D., Surgeon to the Pennsylvania Hospital.

Conjunctival polypi are so rarely met with, that even the best illustrated works on Ophthalmic Diseases contain no drawings taken from patients with this affection.

Even the splendid work of Dalrymple, replete as it is with beautifully executed engravings, is deficient in this respect.

The standard works of the day—Lawrence, Walton, Jones—have no representation of the kind. Indeed the affection is scarcely alluded to by many writers, and no mention is made of it by others. Walton merely speaks of a case occurring in Mr. Smee's practice.

The subject of this growth came under my notice in 1851, at Wills Hospital. He was a young man about 28 years of age, who applied to the hospital to have a foreign body removed from the eye.

The polypi were two in number, pedunculated and attached to the conjunctiva of the upper lid near the upper margin of the tarsal cartilage. They were soft, moveable, and of a light pink color. The size is well represented in the wood cut. The most pendulous portion of each was somewhat compressed in its antero-posterior diameter. They seemed to have occasioned but little inconvenience to the patient, and it is probable that he would not have presented at the Hospital, had he not been suffering from a piece of straw under the lid. In the ordinary position of the lids they could hardly be observed, but upon everting the lid they appeared as represented in the cut.

They were removed by the scissors without much pain, and the examination of them microscopically exhibited an epitheial covering externally, and a delicate areolar tissue within.

## Figures and Tables

**Figure f1:**